# Protocol for a Randomized Controlled Trial to Determine if Biomarkers Predict Response to a Pediatric Chronic Pain Symptom Management Program

**DOI:** 10.3390/jcm14093185

**Published:** 2025-05-05

**Authors:** Rona L. Levy, Tasha B. Murphy, Margaret M. Heitkemper, Miranda A. L. van Tilburg, Ann R. McMeans, Jocelyn Chang, Cynthia Boutte, Katherine Lamparyk, Bruno P. Chumpitazi, Robert J. Shulman

**Affiliations:** 1 School of Social Work, University of Washington, Seattle, WA 98195, USA; rlevy@uw.edu (R.L.L.); miranda_van_tilburg@med.unc.edu (M.A.L.v.T.); 2 School of Nursing, University of Washington, Seattle, WA 98195, USA; heit@uw.edu; 3 Cape Fear Valley Medical Center, Fayetteville, NC 23804, USA; 4 Department of Psychiatry, University of North Carolina at Chapel Hill, Chapel Hill, NC 27514, USA; 5 Children’s Nutrition Research Center, Baylor College of Medicine, Houston, TX 77030, USA; armcmeans@gmail.com (A.R.M.); cboutte@bcm.edu (C.B.); jocelyn.chang@bcm.edu (J.C.); rshulman@bcm.edu (R.J.S.); 6 Department of Pediatrics, Baylor College of Medicine, Houston, TX 77546, USA; 7 Department of Psychiatry and Behavioral Health, Akron Children’s Hospital, Akron, OH 44308, USA; klamparyk@akronchildrens.org; 8 Duke Clinical Research Institute, Durham, NC 27701, USA; bruno.chumpitazi@duke.edu

**Keywords:** functional gastrointestinal disorders, disorders of gut–brain interaction, abdominal pain, child, parent, psychosocial intervention, cognitive behavioral therapy, low-FODMAP diet, biomarkers, personalized medicine (or precision medicine)

## Abstract

**Background/Objectives**: Disorders of gut–brain interaction (DGBI), characterized by chronic abdominal pain and significant disability, affect 15–20% of children and adults and continue into adulthood in ~60% of cases. Costs for adults reach USD 30 billion per year, yet effective management strategies are elusive. Studies support using cognitive behavioral therapy (CBT), but abdominal pain only improves in ~40% of patients. Dietary management (low FODMAP diet; LFD) has also shown promise but it is effective in only a similar percentage of patients. Studies suggest that biologic factors (biomarkers) contribute to CBT response. Similarly, gut microbiome composition appears to influence abdominal pain response to the LFD. However, no previous CBT trials in children or adults have measured these biomarkers, and it is unclear which patients respond best to CBT vs. LFD. **Methods**: Children aged 7–12 years with DGBIs (*n* = 200) will be categorized as having/not having Autonomic Nervous System imbalance and/or abnormalities in gut physiology. We will randomize these children to either CBT or a LFD to compare the effectiveness of these treatments in those with/without abnormal physiologic biomarkers. We hypothesize that CBT will be more effective in those without abnormal physiology and LFD will be more effective in children with abnormal physiology. Primary outcome measures include the following: (1) Symptom improvement (abdominal pain frequency/severity) and (2) improvement in health-related quality of life. **Conclusions**: This innovative multidisciplinary study is the first to identify physiological characteristics that may moderate the response to two different management strategies. Identification of these characteristics may reduce the burden of these disorders through timely application of the intervention most likely to benefit an individual patient.

## 1. Introduction

Disorders of gut–brain interaction (DGBI), also known as functional gastrointestinal disorders, are a group of disorders characterized by chronic gastrointestinal (GI) symptoms with an absence of underlying pathophysiology [1,2]. DGBI are classified based on symptom-based Rome IV criteria [3] and include pediatric disorders such as irritable bowel syndrome (IBS), functional abdominal pain (FAP), and functional dyspepsia (FD). These disorders are associated with chronic abdominal pain ranging from mild to severe (often with significant disability), altered stooling pattern, and psychological characteristics (including anxiety, depression, and impaired health-related quality of life) [4,5,6,7]. Additionally, pathophysiology (e.g., impaired gut barrier function, low-grade mucosal inflammation) appears to contribute to these disorders [8].

DGBI affect up to 15–20% of school-age children and adults worldwide [9,10]. Childhood DGBI continue into adulthood in many cases [11]. Despite the ubiquity of DGBI and the tremendous associated economic, social, and emotional burdens, widely effective treatment strategies have been elusive, in part because the definitions of the disorders have changed over time, are symptom-based, and are open to interpretation [12,13,14,15,16]. Thus, studies are needed to determine which pathophysiology and psychosocial factors contribute most to treatment response (or its lack) to a specific intervention. Additionally, identifying factors contributing to symptoms and how they may interact is central to the development of interventions to reduce or alleviate pain and disability, and potentially prevent or ameliorate the progression of these disorders into adulthood [13].

### 1.1. Altered Autonomic Nervous System (ANS) Function

The sympathovagal balance of the ANS plays a major role in regulating GI function including motility, secretion, and gut barrier function [17,18,19]. It has been hypothesized that alterations in the balance of sympathetic and parasympathetic functions, as measured by heart rate variability (HRV) input, contribute to abdominal pain/discomfort, constipation and diarrhea in DGBI [20]. Our group has shown a relationship between decreased HRV and increased depression in children with DGBI; however, it is unclear whether a relationship between HRV in response to CBT exists in children and/or adolescents with FGIDs [21,22,23].

### 1.2. Role of Diet

Studies have shown that 70–90% of children and adults with DGBI state that foods exacerbate their GI symptoms [24,25,26]. Fermentable oligosaccharides disaccharides and polyols (FODMAPs) are poorly absorbed and increase intestinal gas and/or induce an osmotic effect believed to be responsible for provoking symptoms. Data suggest that certain FODMAP sugars also can increase gut permeability and cause gut mucosal inflammation, or increase serum proinflammatory cytokines in IBS [27,28,29,30,31].

The LFD is a dietary intervention that restricts FODMAP dietary intake and has been shown to improve symptoms in adults and children with DGBI [32,33,34,35]. Pain remits within a few days of starting an LFD, with ~50% of pediatric and adult patients ultimately responding [35,36,37,38]. Our team has found that a diet eliminating a number of FODMAP foods in children with IBS resulted in only 30% of 113 children having a ≥50% improvement in abdominal pain frequency [39]. Importantly, psychosocial factors in both our randomized, double-blind FODMAP and fiber supplementation trials did not predict response [36,39].

### 1.3. Altered GI Microbiome Composition

Studies also suggest that the composition of the GI microbiome differs between DGBI patients and healthy individuals [40,41]. In a small group of children with IBS (*n* = 22), composition was related to severity of abdominal pain symptoms; this parallels findings in adults with DGBI [40,41,42,43,44,45,46,47]. Visceral hyperalgesia is a common finding in children and adults with DGBI that may be related to GI microbiome composition via the upregulation of immune responses, which, in turn, can increase gut permeability. Gut inflammation, the degree of which can be modified by gut bacteria and which can also be gender-dependent, can affect the function of smooth muscle and enteric nerves, resulting in dysmotility [48,49,50,51,52]. These data underscore the potential contribution of the GI microbiome not only to abdominal pain but also to other gut alterations (increased GI permeability and inflammation) found in some patients with DGBI. However, discrepancies exist among studies as to the strength of the relationship between symptoms (abdominal pain, bowel pattern) and differences in the gut microbiome. Thus, the links between microbiome signatures and symptoms of DGBIs remain to be fully elucidated.

The potential relationship between gut microbiome composition and responsiveness to treatment is varied; CBT has received scant attention in the adult and pediatric literature [53,54]. For example, Chumpitazi et al. showed in children that baseline microbiome composition predicted response to an LFD, as well as to a challenge with the FODMAP fructan [55,56]. Discrepancies among studies investigating the potential relationship between gut microbiome composition, symptoms, and psychological distress and response to treatment (CBT, LFD) are likely due to differences in patient populations, bacterial sequencing methodology, and the data analytic approaches used.

### 1.4. Psychosocial Factors

Psychosocial factors have long been felt to be an important contributor to childhood/adult DGBIs. Stress and parental modeling and responses have been proposed to account for the maintenance of pain and disability [57,58,59]. Parental and child cognitions about the origin, severity and controllability of the disorder (e.g., catastrophizing) in particular have been associated with increased pain severity and disability, suggesting that when parents and children perceive pain to be uncontrollable and threatening, the child likely will have worse outcomes [60]. Our novel observation that psychosocial factors are greater in children with DGBI who have normal vs. increased gut permeability supports the idea that gut pathophysiologic changes and psychosocial factors may provoke symptoms independently. To permit appropriately targeted DGBI interventions, these data highlight the critical need to investigate in individual patients whether pathophysiologic changes are present, and if so, whether they exist independently or are interactive with psychological factors.

### 1.5. Use of Cognitive Behavioral Therapy (CBT)

Meta-analyses of adult and pediatric studies support the use of cognitive behavioral therapy (CBT) and associated modalities as our best strategy to treat DGBI pain [61,62,63]. CBT is thought to improve patient pain reports, distress and function by altering dysfunctional cognitions and increasing patient involvement in activities through behavioral management, such as decreasing positive consequences for illness behaviors and increasing support for wellness behaviors and the use of coping strategies (e.g., relaxation and distraction). However, little is known about which patients are more likely to respond and which others likely require different management (e.g., dietary management, probiotics) [64,65].

In adults and children, abdominal pain only improves in ~40% of patients with CBT, in some cases delaying/supplanting more targeted management [66]. A recent Cochrane meta-analysis demonstrated that CBT was an effective treatment for reducing pain intensity in children with DGBI (OR 5.67, 95% CI 1.18–27.32), with more data supporting this management strategy than for other psychosocial interventions [67]. However, there was a dearth of follow-up at 3 months [67]. These findings match those from a previous systematic review [62]. The Cochrane meta-analysis, as well as one by Fisher et al., found insufficient evidence for an effect of CBT on disability [67,68]. Such findings have led to recommendations for research in pediatric patients to further establish the efficacy of CBT for non-pain outcomes such as disability, and to test CBT efficacy delivered via other change agents (i.e., parents) [61].

Our group has developed and tested an intervention for children with DGBI and their parents focused on modifying child and parent cognitions and behavioral responses to pain behaviors, with resulting reductions in child GI symptoms and improvements in functioning (i.e., fewer missed school days) [69]. A mediation analysis suggested that changes in child/parental cognitions and parental responses to their child’s pain mediated these positive outcomes. Thus, we conducted a subsequent study demonstrating that an intervention delivered via telephone to parents alone also was effective in improving child functioning [70]. In other studies, disability (including school absences/healthcare utilization) has been shown to be influenced by coping, independent of pain level, and coping (catastrophizing) has been found to mediate CBT response [71,72,73].

Our own work and that of others shows that some effects are durable for at least 12 months [74,75]. However, we found that ~50% of participants in our initial RCT of CBT for DGBI showed a <10% improvement in abdominal pain or other GI symptoms at 6-month follow-up [69,74]. While this may be due, in part, to the fact that baseline levels of pain and symptoms were overall relatively low, this finding also suggests the possibility that a lack of treatment response to CBT could occur in a subset of children with DGBI related to one or more physiological abnormalities described above—a question this study will address. It may be that modest effect sizes in this and other studies of CBT for DGBI are partly the result of unrecognized GI pathophysiology limiting CBT effectiveness—a knowledge gap this study seeks to fill [64]. Failure to recognize the presence of these physiological factors could result in delays in initiating other potentially effective therapies (e.g., dietary management). Given that factors consistently predictive of response to CBT for pediatric abdominal pain have not been identified [76], studies such as the one proposed here that simultaneously examine psychosocial factors and pathophysiology are likely to provide novel insight into predictors of CBT response.

### 1.6. Summary

The use of biomarkers to distinguish those who would benefit from CBT vs. more from an alternative treatment is innovative, and could assist healthcare providers in selecting appropriate treatment for an individual patient, particularly as there are no drugs clearly shown to be effective for treating pediatric DGBI [77]. The widely accepted premise that etiology and/or maintenance of DGBI is best understood within a biopsychosocial model incorporating interactions among biological/psychological/environmental factors offers potential direction in treatment decisions [78,79]. In an individual patient, one or more physiologic factors (e.g., gut dysbiosis, barrier dysfunction) may contribute more to symptom generation than in another patient with only psychosocial distress, yet studies have paid insufficient attention to physiologic factors potentially predicting poor response to CBT; a critical gap [65,80]. To personalize treatments, data are needed delineating factors affecting responsiveness.

## 2. Materials and Methods

### 2.1. Trial Design

**Figure 1:**
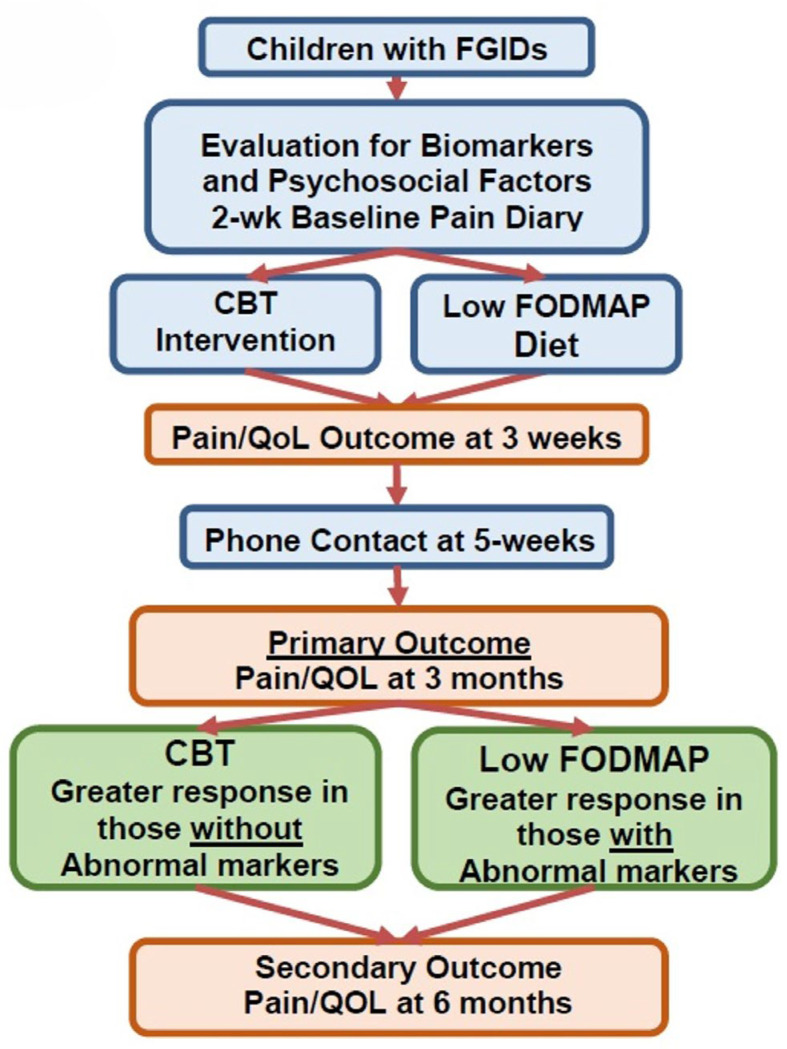
Study Recruitment Flow and Condition Allocation.

The proposed study is a randomized controlled trial with a two-group design (CBT vs. LFD). Figure 1 shows the study recruitment flow and condition allocation.

### 2.2. Study Setting

Children with DGBI will be recruited through the Texas Children’s Hospital Pediatric Gastroenterology, Hepatology, and Nutrition Service (4 nurse practitioners, 37 physicians) and the Texas Children’s Hospital Primary Care Associates (TCPA), which is the nation’s largest group of general pediatricians (50 nurse practitioners, 205 physicians) at 50 locations throughout the Houston metropolitan area.

### 2.3. Eligibility Criteria

The sample will include 200 children with DGBI. Complete inclusion and exclusion criteria are presented in Table 1.

### 2.4. Sample Size

The sample size will be 100 subjects per treatment group (i.e., 200). Table 2 shows how power depends on effect size, which is unknown, and also the fraction of subjects in the abnormal biomarker subgroup. Power for detecting an interaction should be good if the effect size is ≥0.9. In our prior study delivering CBT to adults with DGBI and measuring biomarkers (*n* = 85), the effect sizes observed were 1.12 for LF/HF ratio and 0.76 for high-frequency HRV [81]. Given our proposed sample size is >2-fold greater than in our previous studies and 30% of children with DGBIs have increased gut permeability [8], we should be able to detect potential differences between groups.

### 2.5. Recruitment

Children with DGBI will be recruited through the Texas Children’s Hospital Pediatric Gastroenterology, Hepatology, and Nutrition Service and the TCPA, as described above. Thus, the profile of the local population will be reflected in the study sample (see Enrollment Report). Our findings should be generalizable to the real-world situation as we will recruit from primary and tertiary care. IRB approval will be obtained. Subjects will be identified by reviewing billing records for ICD-10 codes for abdominal pain and IBS (DGBIs). All medical charts will be reviewed by one of the principal investigators (Dr. Shulman). Families will be invited to participate in a letter sent out by their nurse practitioner, pediatrician, or pediatric gastroenterologist. All healthcare providers are in the same academic practice, so there is full participation in referring children to the study. Once the letter is sent out, the research coordinators will call the family for additional screening using the questionnaire developed by the Pediatric Rome IV Working Group [3].

### 2.6. Randomization

Group allocation will be determined using a computer-generated algorithm with equal assignments to each study arm. Subjects will be stratified based on age (7–9 vs. 10–12), sex, baseline pain (mean pain frequency (≤50% of the rated intervals or >50% of the rated intervals, as reported on the baseline 2-week pain diary)), QOL, and presence/absence of abnormal physiological biomarkers.

### 2.7. Intervention

#### 2.7.1. Cognitive Behavioral Therapy (CBT)

Study treatments will be conducted by psychologists or master’s-level therapists with a minimum of 2 years of CBT postgraduate experience. They will receive at least 30 h of training in the manualized therapy prior to conducting treatment sessions. Training will consist of didactic presentations, modeling, role-playing by therapists, and corrective feedback, with repetition as needed. Therapists will be provided with a standardized protocol for each session. Treatment sessions also will be audio-recorded.

CBT treatment will be manualized and consist of a 60 min session conducted by phone once per week for 3 weeks. The number of treatment sessions was based on the successful outcomes of our previous studies and our commitment to developing treatments that are maximally accessible [69,70,81,82,83]. The intervention will be delivered to parents via telephone. Intervention strategies that involve children, such as at-home distraction or relaxation exercises, will be taught to children by their parents. Based on our work described above and that of others [69,84,85], the intervention has the following primary goals: Teaching parents a.) ways to differentially encourage, attend to and reinforce wellness behaviors (those behaviors incompatible with illness and disability) while decreasing attention to and reinforcement of illness behaviors related to abdominal pain; (b) more adaptive cognitive coping strategies and perspectives on their child’s pain, including reducing catastrophizing cognitions and threat appraisals regarding their child’s DGBI; (c) techniques for coaching their child to engage in cognitive-behavioral coping relaxation strategies, stress management and cognitive techniques (e.g., examining and altering self-talk); (d) how to model healthy responses to somatic symptoms; and (e) enabling parents to teach their children strategies such as relaxation training by providing parents audio-recordings to utilize with their children. Van Tilburg et al. has shown that guided imagery, a common relaxation technique applied within CBT, can be successfully delivered through audio-recordings to children with DGBI [86]. The intervention is specifically targeted to improve coping and management skills for symptoms, decrease healthcare utilization and functional disability, and improve QOL. Treatment fidelity—the extent to which a therapist uses interventions prescribed by a protocol [87]—will be closely monitored.

#### 2.7.2. Low FODMAP Diet Condition (LFD)

There are three phases in an LFD. (1.) Restriction phase: strict avoidance of high-FODMAP foods, and replacing them with low FODMAP alternatives; if symptoms improve, one can move to the next phase. (2.) Reintroduction phase: gradually reintroduce each FODMAP to determine which FODMAP(s) and at what doses trigger symptoms. (3.) Personalization phase: continued monitoring of symptoms with tailored diet for long-term adherence and nutritional adequacy [88,89,90].

The LFD will be administered by registered and licensed dietitians due to the risks of nutritional inadequacy and disordered eating. Parents will receive detailed instructions for following an LFD with recommendations for helping their children follow and adhere to the diet. They will be taught the definition of FODMAPs and how to identify and select low-FODMAP foods, and will be provided with tools they can use to help with diet compliance. Families will also be provided with recipes and a table detailing foods to avoid and foods allowed on the diet, as well as some low-FODMAP foods (such as gluten-free pasta and bread and low-FODMAP sauces) to help them get started. Recommendations will be tailored based on the child’s likes and dislikes, foods the family normally eats, and special circumstances (i.e., holidays and vacations). All 3 phases of the LFD (described above) will be implemented, with special focus on the elimination phase, in a similar fashion to other studies [91]. The dietitian will contact the family for approximately 1 h once a week for three weeks to check on the progress of following the diet, provide additional information as needed, and answer any questions. Thus, the CBT and low-FODMAP groups will have a similar degree of contact (once weekly for 1 h) with the study staff. As in the CBT group, sessions will be audio-recorded and reviewed for protocol consistency. Parents will complete a food diary at baseline and at post-treatment (while on the LFD; post-treatment food records will be used to determine adherence to the LFD). The Nutrition Data System for Research (University of Minnesota) will be used to analyze the food diaries. Based on our previous studies, compliance with the diet is >90% [36,38,92]. The children will have FODMAPs reintroduced gradually into their diet under the guidance of the dietitian, as is done clinically. Should a specific FODMAP appear to cause abdominal pain, the child may continue to exclude that food from their diet.

### 2.8. Outcomes

Primary outcomes are abdominal pain and QOL at 3 months. Secondary outcomes include pain and QOL at 6 months, healthcare utilization, missed school days, and sleep.

### 2.9. Study Timeline

The study timeline includes an enrollment period, baseline assessment, randomization, treatment, and 5 post-treatment assessment periods (immediately post-treatment, and 3,6,12, and 18-months post-treatment), as shown in Table 3. Specific measurements and timepoints are shown in Table 4.

### 2.10. Blinding

With the exception of study coordinators, therapists, dietitians, and statisticians, all other study staff will be blinded to treatment assignment. Blinding will be made possible through restricting access to participants’ treatment assignments in the study database only to staff whose role is to assign participants to interventions, contact participants throughout the intervention phase, deliver study treatments, or analyze results by treatment group. Study participants will also not be blinded to treatment.

### 2.11. Measures

All self-report parent and child measures were administered using Research Electronic Data Capture (REDCap) electronic data capture tools [93,94] hosted at the University of Washington. A list of measures with descriptions and timings of assessments can be found below and in Table 4.

#### 2.11.1. Demographic Questionnaire

A demographic questionnaire created by our team asked questions about parent and child age, gender, ethnicity, and race. Parent education, marital status, employment, and household composition were also assessed. The questionnaire also asked about child health insurance, number of languages spoken, child birth history, breastfeeding history, number of child’s siblings, % of food the child eats that are organic and genetically modified, child height and weight, and child education (grade in school, type of school, and school enrollment).

#### 2.11.2. Youth/Adolescent Questionnaire (Diet Diary)

At the time of the stool collection, all participants will complete a 3-day diet diary using the Youth/Adolescent Questionnaire validated in this age group [95]. This questionnaire asks parents to record everything their child ate and drank each day (name of food, quantity/amount consumed, how prepared, where prepared [restaurant vs. home] and time of eating). Responses were reviewed by the study dietitians who asked parents for further clarification when needed.

#### 2.11.3. Pain and Stool Diary (Primary Outcome Measure: Pain Frequency and Severity)

A 14-day pain and stool diary will be completed by the child with help from their parent. The diary asks whether pain was experienced each day. If yes, additional questions are asked about pain severity (rated using a numeric rating scale (0–10, with 0 = “none” and 10 = “all the time”) that has been validated for measuring abdominal pain in children) [96,97], and pain frequency (how many times pain occurred). For morning, afternoon, and evening pain, questions are asked about pain duration, pain location, and interference with activities. Stooling questions (how many times child passed stool each day, stool consistency [Bristol Stool Chart], timing of stools, and whether pain occurred with stool) are also included. Additional questions (0–10 scale, with 0 = “none” and 10 = “all the time”) include things that happen while eating or right after eating (nausea, feeling full, burping, and bloating), as well as other symptoms (pain or burning above the belly button, passing gas, heartburn, fatigue, and headache).

#### 2.11.4. Pediatric Quality of Life Inventory (PedsQL; Primary Outcome Measure)

Health-Related Quality of Life (HRQOL) will be assessed using the Pediatric Quality of Life Inventory (PedsQL) 4.0 generic core scales [98,99,100]. The PedsQL is the most widely used measure of pediatric HRQOL, consisting of 23 items. Items are scored on a 5-point Likert scale ranging from 0 (never a problem) to 4 (almost always a problem). Total scores range from 0 to 100 with 100 indicating excellent HRQOL. A psychosocial health summary score also is calculated as a sum of the emotional, social, and school functioning subscales. The generic core scales have adequate feasibility, reliability, and validity in children with DGBIs [7,99].

#### 2.11.5. Children’s Sleep Habits Questionnaire (CSHQ; Secondary Outcome Measure)

Sleep problems will be screened using the 45-item Children’s Sleep Habits Questionnaire (child and parent form), one of the most widely-used and validated tools to screen for pediatric sleep problems in school-age children [101]. Sleep behavior is measured on 33 items scored as usually (5–7X/week), sometimes (2–4X/week), and rarely (0–1X/week). A total score ≥ 41 discriminates between children with/without sleep problems (sensitivity 0.80; specificity 0.72) [101].

#### 2.11.6. Healthcare Utilization and Missed School Days (Secondary Outcome Measures)

Parents will be asked to retrospectively provide the number of visits to a healthcare provider for abdominal pain and/or any illness. Increased school absenteeism is common in children with DGBI. Thus, missed school days often are used as a measure of functional disability [102]. Parents will retrospectively provide the number of school days and other activities missed by their child in the previous 3 months due to pain and/or illness.

#### 2.11.7. Heart Rate Variability (HRV)

Ambulatory electrocardiogram Holter monitors will be used to measure HRV over 20 min while the children are lying quietly [23,81,103,104,105]. All HRV measurements will be obtained at the same time of day. GE Healthcare Holter 3-channel SEER 1000 Recorders (GE Healthcare, Wauwatosa, WI) will be used, and the results will be processed using GE CardioDay 2.5 software.

#### 2.11.8. GI Permeability

All participants will undergo GI permeability testing [8,106]. Participants will fast overnight and urinate upon arising. They will then drink a 100 mL solution containing lactulose (5 g) and mannitol (1 g) followed by 240 mL of water after which time they fast for 4 h. Urine will be collected hourly for the first 4 h and then all urine passed between 4 and 24 h will be combined and analyzed (increased urinary lactulose/mannitol ratio indicates increased small intestinal permeability) [107,108,109].

#### 2.11.9. Gut Microbial Composition

A stool sample will be collected as we have described using a container that seals and serves as its own storage system, so the handling of the stool is not required (Fischer Scientific Inc., Pittsburgh, PA, USA) [39,110]. Bacterial DNA will be extracted using the PowerSoil DNA Isolation kit (MO BIO Laboratories, Carlsbad, CA, USA), with the Human Microbiome Project’s modifications to the manufacturer’s protocol [111]. Whole Genome Shotgun (WGS) metagenomic sequencing will allow the identification of bacterial taxa (species) as well as their metabolic functional potential. Although samples will be sequenced at various time points throughout the study (and thus, on multiple sequencing runs), repeated sequencing of the same specimens has shown, in our hands, to result in >99% concordance between taxonomic profiles (unpublished data). All sequence data will be deposited in the NCBI dbGaP, and detailed sequence analysis protocols will be provided in all related publications.

#### 2.11.10. Fecal Granins

Stool collected for microbiome analysis also will be used to measure chromogranin A and secretogranin III—the granins our preliminary data suggest are elevated in children (as in adults) with DGBIs, and which correlate with abdominal pain symptoms [112,113].

#### 2.11.11. Pain Response Inventory (PRI)

The Pain Response Inventory [114] consists of 31 items that assess children’s coping responses to recurrent pain. It assesses 3 broad coping factors (Active, Passive, and Accommodative). Items are scored on a 0–4 scale (0 = never to 4 = always). It has been validated in pediatric samples and has demonstrated good internal consistency and reliability [114].

#### 2.11.12. Symptom Checklist 90-Revised (SCL-90-R)

The Symptom Checklist 90-Revised is a widely used questionnaire for measuring a range of psychological and psychiatric symptoms [115,116]. It involves 9 dimensions, including somatization, depression, and anxiety. It has been validated in child and adolescent populations, and has demonstrated acceptable internal and test–retest reliability.

#### 2.11.13. Pain Catastrophizing Scale (PCS)

The Pain Catastrophizing Scale [117,118] measures dimensions of rumination, magnification and helplessness that are subsumed under the higher-order construct of pain catastrophizing. The PCS has been validated in children and adolescents, including those with chronic pain, and has demonstrated acceptable reliability [119,120].

#### 2.11.14. Pain Behavior Checklist (PBCL)

The 17-item Pain Behavior Checklist [121] measures pain behaviors across four domains: distorted ambulation, affective distress, facial/audible expressions, and seeking help. The PBCL has been used in prior studies of children with functional abdominal pain and has adequate reliability [70].

#### 2.11.15. Tanner Stage Questionnaire

The Tanner Stage Questionnaire [122,123] assesses the physical development of children into adolescence and then adulthood. It defines physical measurements of development based on primary and secondary sex characteristics, indicating pubertal stages.

#### 2.11.16. Behavior Assessment System for Children-3 (BASC-3)

The BASC-3 [124] includes both parent and child/adolescent self-report of a child’s emotional and behavioral functioning. Item responses are converted into summary scales and indexes. For the parent report, there are two summary scales (clinical and adaptive scales, including anxiety, attention problems, depression, and somatization; content scales include anger, emotional self-control, executive functioning, and resiliency) and two indexes (clinical index includes ADHD probability, clinical probability, and functional impairment; executive functioning index includes attentional, behavioral, and emotional control, and problem-solving). Composite scores include behavioral symptoms, externalizing problems, internalizing problems, and school problems. For the child report, there are two summary scales (clinical and adaptive scales, including anxiety, depression, interpersonal relations, self-esteem, and somatization; content scales include anger control, ego strength, and mania), one index (functional impairment), and composite scores (includes emotional symptoms, internalizing problems, and a total score). Validity and reliability have been well established [124,125].

#### 2.11.17. Treatment Credibility Scale

Treatment credibility will be assessed with a brief survey created by our team. Items include questions about how confident the parent is that this treatment will be successful in reducing their child’s abdominal pain, if they would recommend this treatment to a friend, and if treatment would help them manage their child’s abdominal pain. Items range from 0 (“not at all”) to 9 (“very much”).

#### 2.11.18. Sleep Self Report (SSR)

The child-report Sleep Self Report is a 26-item retrospective survey that assesses the frequency of occurrence of constructs such as bedtime resistance, night awakenings, sleep duration, and daytime sleepiness in the past week [126]. Each item is rated on a 3-point response scale (rarely, sometimes, usually) with a higher score indicating more sleep problems. Items are summed for a total score. Internal consistency and reliability has been reported at 0.88 [126].

#### 2.11.19. ROME-IV Diagnostic Questionnaire on Pediatric Functional Gastrointestinal Disorders (R4PDQ)

The R4PDQ includes child self-report and parent proxy-report forms assessing presence, frequency, and severity of gastrointestinal (GI) symptoms (including pain, bloating, stooling, nausea/vomiting) [127]. Item response scales are varied—some items have a simple “yes/no” response option, whereas others are on a Likert Scale (“never” to “always”). Item scoring derives summary scores that indicate whether a child meets criteria for various functional GI disorders including Functional Dyspepsia, Irritable Bowel Syndrome, and Functional Abdominal pain.

#### 2.11.20. Adults’ Responses to Children’s Symptoms (ARCS; Protect and Minimize Only)

Parental solicitousness in response to pain behavior will be measured using the Protectiveness subscale of the Adults’ Responses to Children’s Symptoms [128]. All 11 items are rated on a 0–4 scale, with higher values indicative of greater solicitousness.

#### 2.11.21. Pain Beliefs Questionnaire (PBQ)

The Pain Beliefs Questionnaire [129,130] 20-item threat scale includes items assessing the perceived duration, frequency, and seriousness of the abdominal pain condition, as well as the intensity and duration of individual pain episodes. Items are rated on a 0–4 scale (0 = not at all true to 4 = very true).

#### 2.11.22. Functional Disability Inventory (FDI)

The Functional Disability Inventory [131] measures the degree to which children experience difficulties in physical and psychosocial functioning. Respondents are asked to rate how much physical difficulty they experienced doing everyday activities such as walking up stairs or doing something with a friend. Responses range from 0 (no trouble) to 4 (impossible). A total score is derived by summing the ratings for each item. Higher scores indicate greater disability. The FDI has been validated in several pediatric populations, including those with chronic pain, and has demonstrated adequate reliability [132,133,134].

### 2.12. Participant Retention and Follow-Up

Participants will receive automated assessment survey invitations and reminders. Research coordinators will send reminders if assessments are not complete, until the completion of the assessment period. We will use a database checklist to ensure that all study procedures are being followed (including consent) and that all surveys are being completed according to schedule. Study staff will review study participant data and monitor parent responses. Participants will also receive gift card incentives for completing baseline and follow-up assessments. All study questionnaires will be completed online so that participants may complete them at a time and place that is convenient for them.

### 2.13. Data Management

All survey data will be collected from parents online using REDCap electronic data capture tools hosted at the University of Washington. REDCap is a secure, web-based software platform designed to support data capture for research studies, providing (1) an intuitive interface for validated data capture; (2) audit trails for tracking data manipulation and export procedures; (3) automated export procedures for seamless data downloads to common statistical packages; and (4) procedures for data integration and interoperability with external sources [95]. Research coordinators will track assessment completion regularly, monitor for any missing data, and follow up with participants when missing data are identified. Research coordinators will also use REDCap alerts, reports, and queries to minimize missing data and ensure accuracy. Additionally, study staff will monitor for data irregularities such as skip patterns and out-of-range data and completion times. The REDCap database will require online sign-in with a username and password assigned to individual study staff; all data stored in REDCap will be retained on a secure server accessible only to study staff. The scoring of study measures will be done via syntax to minimize errors.

### 2.14. Confidentiality

The subject’s identity, research records, and personal health information will be safeguarded using password-protected servers. The primary source of data will be questionnaires, which will be stored electronically in REDCap. All data will be coded with a unique participant ID. The software will be hosted on a secure, HIPAA-compliant server. Only the research team and the Baylor College of Medicine IRB will be able to access the participant data and information collected from this study. All research data will be de-identified at the earliest possible opportunity to promote data sharing.

### 2.15. Statistical Methods

The primary outcome variables at 3 months after completing treatment will be mean number of pain episodes/week and QOL. An analysis of covariance model will be fit, with pain frequency and QOL as the outcome variables, and treatment group (CBT or LFD) and biomarker group (abnormal versus normal) as factors, using baseline pain frequency and QOL as covariates. The interaction between treatment group and biomarker group will be included in the model, and the test of this interaction term will be the primary test of interest. If this term is zero then the null hypothesis is true and the effectiveness of CBT relative to the LFD is the same in the two biomarker groups. If the interaction is significantly different from zero, the relative effectiveness of CBT versus the LFD differs between the two biomarker groups. A general overview of this analytic approach can be found in Figure S1 in the Supplementary Materials. We will test the effect of sex using the above-described analysis of covariance model, wherein sex will be used instead of biomarker group as a factor. Similar analyses will be done for the additional secondary outcome measures of healthcare utilization, missed school days, and sleep. We will examine pain and QOL trajectories. However, 3 months is specified as the primary outcome time because if the effect does not last until at least 3 months, it is less impactful clinically, and there will be fewer missing data at 3 than at 6 months.

Baseline pain frequency/QOL potentially could confound the interaction effect being tested—treatment effectiveness (improvement with CBT minus improvement with the LFD) may be greater for those with higher baseline pain frequency/lower QOL. If so, the interaction between treatment group and baseline pain frequency also will be included in the model.

Two approaches will be considered regarding missing data. The first uses a mixed model to analyze all three time points together. This allows some time points to have missing data, but still gives unbiased estimates if data are missing at random, conditional on the data that are not missing. The second will be multiple imputations, which imputes missing data based on the data not missing.

### 2.16. Adverse Event Reporting

This study has been designated as minimal risk by the study’s Institutional Review Board. Monitoring study safety will occur from the initial screening, throughout the informed consent process, and through study completion under the principal investigators’ supervision. A Safety Monitoring Committee (SMC) consisting of two independent investigators will review study progress and recommend appropriate action regarding adverse events or other safety issues.

In the case of an adverse event believed to be related to the study, documentation will be collected to describe the nature of the event and any associated risks, and event reports will be provided to the IRB and SMC for review.

### 2.17. Dissemination Plan

Study results will be disseminated via publication in peer-reviewed journals, reporting on ClinicalTrials.gov within 12 months of study completion, and presentations at professional society meetings. No identifying images or other personal or clinical details of participants will be presented in reports of the trial results. A summary of the study findings will be sent via email to all study participants after the final trial endpoint completion.

## 3. Results

Study results will be submitted for publication at the conclusion of the trial.

## 4. Discussion

This study will assess the relevance of physiologic biomarkers in determining the efficacy of pediatric DGBI interventions (parental-focused CBT vs. LFD). The results have the potential to address a significant knowledge gap and thus to help determine if the assessed physiologic biomarkers affect outcomes several months after the intervention. Future changes in clinical practice may include testing for the relevant physiologic markers prior to recommending either CBT or the LFD. If the use of biomarkers in clinical practice becomes more common, and reductions in cost follow, they have the potential to be seamlessly integrated into clinical practice treatment for biomarker-based treatment stratification. One significant upside of permeability testing is that it can be done at home. The patient is simply given the materials for collection in the clinic, and then drops the samples off after collection.

The strengths of the study include its randomized controlled trial structure, which will randomize participants and ideally account for known and unknown confounding factors. The proposed questionnaires are validated, and have been used in studies of children with DGBI in the past.

A limitation of the study is that the physiologic markers will not be followed longitudinally past the initial post-treatment assessment. This will preclude evaluations to determine whether changes in physiologic markers are occurring at 3 months that may be more associated with the outcome during that time period. Nonetheless, in an attempt to minimize the burden to subjects, and because the primary aim was to determine the relevance of baseline physiologic factors on subsequent outcomes, the study was designed accordingly.

The results of this project can contribute significantly to the clinical and psychosocial treatment of children with DGBIs. As we have stated, treatment strategies for children with DGBIs have been elusive. CBT and LFD have been explored as potential treatment options, but a critical knowledge gap remains in determining which patients are more likely to respond to psychosocial intervention such as CBT, and which require different management approaches (e.g., dietary management, probiotics). This study will be the first to address the critical need to identify physiological characteristics that may moderate the responses to CBT or LFD interventions. The use of biomarkers in making this determination is innovative, and these results can be applied to reduce the burden of DGBIs through the timely application of the intervention most likely to benefit an individual patient.

## Figures and Tables

**Table 1:** Eligibility criteria.

Inclusion Criteria
Child age 7–12 years Medical evaluation negative for organic cause of abdominal pain and symptoms meeting the definition of a DGBI per pediatric Rome IV criteria. Parent and child speak English
Exclusion Criteria
Children who have had: ○ Past bowel surgery ○ Organic GI disorder ○ Serious chronic medical condition (i.e., diabetes) ○ Weight and/or height < 2 SD for age ○ Chronic condition(s) with GI symptoms (e.g., cystic fibrosis) ○ Autism Spectrum Disorder ○ Significant developmental delay ○ Psychosis and/or bipolar disorder ○ Have been treated with antibiotics/probiotics within 1 month (possible gut microbiome effects) ○ Children who are currently using the LFD or are receiving CBT

**Table 2:** Relationship between power and effect size.

Effect Size *	Power % Fraction ^#^ = 0.5	Power % Fraction = 0.33	Power % Fraction = 0.25
0.6	56	51	45
0.7	69	64	57
0.8	80	75	68
0.9	89	85	78
1.0	94	91	86

* Effect Size = magnitude of the interaction term divided by the within group standard deviation. ^#^ Fraction of subjects who fall within the abnormal group or the normal group, whichever is smaller.

**Table 3:** Schedule of enrollment, interventions, and assessments.

Timepoint	Enrollment	Baseline t1	Randomization	Treatment	Post-Treatment *				
					t2	t3	t4	t5	t6
**Enrollment:**									
Eligibility Screen	X								
Informed Consent	X								
Randomization			X						
**Interventions:**									
CBT				X					
LFD				X					
**Sample Collections:**									
Stool Sample		X			X				
Saliva Sample		X			X				
24 h Urine Collection		X			X				
Heart Rate Monitor (20 min collection)		X			X				
**Assessments:**									
Demographics		X							
Medical History		X							
Biological/Physiological Measures		X			X				
Outcomes Measures		X			X	X	X	X	X
Other Measures		X			X	X	X	X	X

* *t2–t6*: post-intervention assessments (t2, post-treatment; t3, 3 months; t4, 6 months; t5, 12 months; t6, 18 months).

**Table 4:** Measures.

Measure	Baseline	Post-Treatment				
	t1 *	t2 *	t3 *	t4 *	t5 *	t6 *
Demographic Questionnaire	P					
Diet Diary	PC	PC				
**Primary Outcome Measures**						
Pain Frequency and Severity (from 14-day Day Pain and Stool Diary; Child report with help from parents)	C	C	C	C	C	C
Pediatric Quality of Life Inventory (PedsQL)	PC	PC	PC	PC	PC	PC
**Secondary Outcome Measures**						
Children’s Sleep Habits Questionnaire (CSHQ)	P	P				
Healthcare Utilization	P	P	P	P	P	P
Missed School Days	P	P	P	P	P	P
**Biological/Physiological Measures**						
Heart Rate Variability	C	C				
GI Permeability	C	C				
Gut Microbial Composition	C	C				
Fecal Granins	C	C				
**Other Measures**						
Pain Response Inventory (PRI)	C					
Symptom Checklist (SCL-90)	P					
Pain Catastrophizing Scale (PCS)	P					
Pain Behavior Checklist (PBCL)	P					
Tanner Stage Questionnaire	P					
Behavior Assessment System for Children (BASC)	PC					
Treatment Credibility Scale		P				
Sleep Self Report (SSR)	C	C				
ROME-IV Questionnaire	PC		PC	PC	PC	PC
Adults’ Responses to Children’s Symptoms (ARCS; Protect & Minimize subscales only)	P	P	P	P	P	P
Pain Beliefs Questionnaire (PBQ) *	PC	PC	PC	PC	PC	PC
Functional Disability Inventory (FDI)	P	P	P	P	P	P

P = parent report, C = child self-report; * t1 = baseline, t2 = post-treatment, t3 = 3 months post-treatment, t4 = 6 months post-treatment, t5 = 12 months post-treatment, t6 = 18 months post-treatment.

## Data Availability

The study protocol and any datasets or statistical code required to support the protocol will be supplied on request. We will also make de-identified datasets available via a public data repository.

## Supplementary Materials

The following supporting information can be downloaded at: https://www.mdpi.com/article/10.3390/jcm14093185/s1, Figure S1: Analysis Outline Plan for Biomarkers.

## References

[1] Corazziari E. (2004). Definition and epidemiology of functional gastrointestinal disorders. Best Pract. Res. Clin. Gastroenterol..

[2] Fikree A., Byrne P. (2021). Management of functional gastrointestinal disorders. Clin. Med..

[3] Drossman D.A. (2016). Functional Gastrointestinal Disorders: History, Pathophysiology, Clinical Features and Rome IV. Gastroenterology.

[4] Rutten J.M., Benninga M.A., Vlieger A.M. (2014). IBS and FAPS in children: A comparison of psychological and clinical characteristics. J. Pediatr. Gastroenterol. Nutr..

[5] Youssef N.N., Murphy T.G., Langseder A.L., Rosh J.R. (2006). Quality of life for children with functional abdominal pain: A comparison study of patients’ and parents’ perceptions. Pediatrics.

[6] Shelby G.D., Shirkey K.C., Sherman A.L., Beck J.E., Haman K., Shears A.R., Horst S.N., Smith C.A., Garber J., Walker L.S. (2013). Functional abdominal pain in childhood and long-term vulnerability to anxiety disorders. Pediatrics.

[7] Varni J.W., Bendo C.B., Nurko S., Shulman R.J., Self M.M., Franciosi J.P., Saps M., Pohl J.F., Pediatric Quality of Life Inventory (PedsQL) Gastrointestinal Symptoms Module Testing Study Consortium (2015). Health-related quality of life in pediatric patients with functional and organic gastrointestinal diseases. J. Pediatr..

[8] Shulman R.J., Eakin M.N., Czyzewski D.I., Jarrett M., Ou C.N. (2008). Increased gastrointestinal permeability and gut inflammation in children with functional abdominal pain and irritable bowel syndrome. J. Pediatr..

[9] Saps M., Seshadri R., Sztainberg M., Schaffer G., Marshall B.M., di Lorenzo C. (2009). A prospective school-based study of abdominal pain and other common somatic complaints in children. J. Pediatr..

[10] Rosen J.M., Saps M. (2014). British secondary school students report frequent abdominal pain with associated physical and emotional symptoms. Evid.-Based Nurs..

[11] Hyams J.S., Di Lorenzo C., Saps M., Shulman R.J., Staiano A., van Tilburg M. (2016). Functional Disorders: Children and Adolescents. Gastroenterology.

[12] Gwee K.A., Ghoshal U.C. (2010). The Rome criteria divides, distorts and dilutes the prevalence of irritable bowel syndrome. Saudi J. Gastroenterol..

[13] Barbara G., Stanghellini V. (2009). Biomarkers in IBS: When will they replace symptoms for diagnosis and management?. Gut.

[14] Camilleri M., Talley N.J. (2004). Pathophysiology as a basis for understanding symptom complexes and therapeutic targets. Neurogastroenterol. Motil..

[15] Moayyedi P., Ford A.C. (2011). Symptom-based diagnostic criteria for irritable bowel syndrome: The more things change, the more they stay the same. Gastroenterol. Clin. N. Am..

[16] Czyzewski D.I., Lane M.M., Weidler E.M., Williams A.E., Swank P.R., Shulman R.J. (2011). The interpretation of Rome III criteria and method of assessment affect the irritable bowel syndrome classification of children. Aliment. Pharmacol. Ther..

[17] Browning K.N., Travagli R.A. (2014). Central nervous system control of gastrointestinal motility and secretion and modulation of gastrointestinal functions. Compr. Physiol..

[18] Rubio A., Van Oudenhove L., Pellissier S., Ly H.G., Dupont P., Lafaye de Micheaux H., Tack J., Dantzer C., Delon-Martin C., Bonaz B. (2015). Uncertainty in anticipation of uncomfortable rectal distension is modulated by the autonomic nervous system--a fMRI study in healthy volunteers. Neuroimage.

[19] Chalaye P., Goffaux P., Bourgault P., Lafrenaye S., Devroede G., Watier A., Marchand S. (2012). Comparing pain modulation and autonomic responses in fibromyalgia and irritable bowel syndrome patients. Clin. J. Pain.

[20] Mroz M., Czub M., Brytek-Matera A. (2022). Heart Rate Variability—An Index of the Efficacy of Complementary Therapies in Irritable Bowel Syndrome: A Systematic Review. Nutrients.

[21] Semen M., Lychkovska O., Kaminskyy D., Yavorskyi O., Semen K.O., Yelisyeyeva O. (2023). Heart Rate Variability and Somatization in Adolescents with Irritable Bowel Syndrome. J. Neurogastroenterol. Motil..

[22] Stern M.J., Guiles R.A., Gevirtz R. (2014). HRV biofeedback for pediatric irritable bowel syndrome and functional abdominal pain: A clinical replication series. Appl. Psychophysiol. Biofeedback.

[23] Jarrett M., Heitkemper M., Czyzewski D., Zeltzer L., Shulman R.J. (2012). Autonomic nervous system function in young children with functional abdominal pain or irritable bowel syndrome. J. Pain.

[24] Carlson M.J., Moore C.E., Tsai C.M., Shulman R.J., Chumpitazi B.P. (2014). Child and parent perceived food-induced gastrointestinal symptoms and quality of life in children with functional gastrointestinal disorders. J. Acad. Nutr. Diet..

[25] Reed-Knight B., Squires M., Chitkara D.K., van Tilburg M.A. (2016). Adolescents with irritable bowel syndrome report increased eating-associated symptoms, changes in dietary composition, and altered eating behaviors: A pilot comparison study to healthy adolescents. Neurogastroenterol. Motil..

[26] Monsbakken K.W., Vandvik P.O., Farup P.G. (2006). Perceived food intolerance in subjects with irritable bowel syndrome—Etiology, prevalence and consequences. Eur. J. Clin. Nutr..

[27] Bovee-Oudenhoven I.M., ten Bruggencate S.J., Lettink-Wissink M.L., van der Meer R. (2003). Dietary fructo-oligosaccharides and lactulose inhibit intestinal colonisation but stimulate translocation of salmonella in rats. Gut.

[28] Ten Bruggencate S.J., Bovee-Oudenhoven I.M., Lettink-Wissink M.L., Van der Meer R. (2003). Dietary fructo-oligosaccharides dose-dependently increase translocation of salmonella in rats. J. Nutr..

[29] Fritscher-Ravens A., Schuppan D., Ellrichmann M., Schoch S., Rocken C., Brasch J., Bethge J., Bottner M., Klose J., Milla P.J. (2014). Confocal endomicroscopy shows food-associated changes in the intestinal mucosa of patients with irritable bowel syndrome. Gastroenterology.

[30] Yang J., Fox M., Cong Y., Chu H., Zheng X., Long Y., Fried M., Dai N. (2014). Lactose intolerance in irritable bowel syndrome patients with diarrhoea: The roles of anxiety, activation of the innate mucosal immune system and visceral sensitivity. Aliment. Pharmacol. Ther..

[31] Narayana V., McMeans A.R., Levy R.L., Shulman R.J., Chumpitazi B. (2023). Children with Functional Abdominal Pain Disorders Successfully Decrease FODMAP Food Intake on a Low FODMAP diet with Modest Improvements in Nutritional Intake and Diet Quality. Neurogastroenterol. Motil..

[32] Chumpitazi B.P., McMeans A.R., Vaughan A., Ali A., Orlando S., Elsaadi A., Shulman R.J. (2018). Fructans exacerbate symptoms in a subset of children with irritable bowel syndrome. Clin. Gastroenterol. Hepatol..

[33] El Ezaby S.A., Manzour A.F., Eldeeb M., El Gendy Y.G., Abdel Hamid D.A. (2024). Effect of the Low Fermentable Oligosaccharides, Disaccharides, Monosaccharides, and Polyols (FODMAP) Diet on Control of Pediatric Irritable Bowel Syndrome and Quality of Life Among a Sample of Egyptian Children: A Randomized Controlled Clinical Trial. Cureus.

[34] Dogan G., Yavuz S., Aslantas H., Ozyurt B.C., Kasirga E. (2020). Is low FODMAP diet effective in children with irritable bowel syndrome?. North Clin. Intanb..

[35] Halmos E.P., Power V.A., Shepherd S.J., Gibson P.R., Muir J.G. (2014). A diet low in FODMAPs reduces symptoms of irritable bowel syndrome. Gastroenterology.

[36] Chumpitazi B.P., Cope J.L., Hollister E.B., Tsai C.M., McMeans A.R., Luna R.A., Versalovic J., Shulman R.J. (2015). Randomised clinical trial: Gut microbiome biomarkers are associated with clinical response to a low FODMAP diet in children with irritable bowel syndrome. Aliment. Pharmacol. Ther..

[37] McIntosh K., Reed D.E., Schneider T., Dang F., Keshteli A.H., De Palma G., Madsen K., Bercik P., Vanner S. (2017). FODMAPs alter symptoms and the metabolome of patients with IBS: A randomised controlled trial. Gut.

[38] Chumpitazi B.P., Hollister E.B., Oezguen N., Tsai C.M., McMeans A.R., Luna R.A., Savidge T.C., Versalovic J., Shulman R.J. (2014). Gut microbiota influences low fermentable substrate diet efficacy in children with irritable bowel syndrome. Gut Microbes.

[39] Shulman R.J., Hollister E.B., Cain K., Czyzewski D.I., Self M.M., Weidler E.M., Devaraj S., Luna R.A., Versalovic J., Heitkemper M. (2017). Psyllium Fiber Reduces Abdominal Pain in Children With Irritable Bowel Syndrome in a Randomized, Double-Blind Trial. Clin. Gastroenterol. Hepatol..

[40] Saulnier D.M., Riehle K., Mistretta T.A., Diaz M.A., Mandal D., Raza S., Weidler E.M., Qin X., Coarfa C., Milosavljevic A. (2011). Gastrointestinal microbiome signatures of pediatric patients with irritable bowel syndrome. Gastroenterology.

[41] Simren M., Barbara G., Flint H.J., Spiegel B.M., Spiller R.C., Vanner S., Verdu E.F., Whorwell P.J., Zoetendal E.G., Rome Foundation C. (2013). Intestinal microbiota in functional bowel disorders: A Rome foundation report. Gut.

[42] Amaral F.A., Sachs D., Costa V.V., Fagundes C.T., Cisalpino D., Cunha T.M., Ferreira S.H., Cunha F.Q., Silva T.A., Nicoli J.R. (2008). Commensal microbiota is fundamental for the development of inflammatory pain. Proc. Natl. Acad. Sci. USA.

[43] Eutamene H., Lamine F., Chabo C., Theodorou V., Rochat F., Bergonzelli G.E., Corthesy-Theulaz I., Fioramonti J., Bueno L. (2007). Synergy between Lactobacillus paracasei and its bacterial products to counteract stress-induced gut permeability and sensitivity increase in rats. J. Nutr..

[44] Wilson M., Seymour R., Henderson B. (1998). Bacterial perturbation of cytokine networks. Infect. Immun..

[45] Crouzet L., Gaultier E., Del’Homme C., Cartier C., Delmas E., Dapoigny M., Fioramonti J., Bernalier-Donadille A. (2013). The hypersensitivity to colonic distension of IBS patients can be transferred to rats through their fecal microbiota. Neurogastroenterol. Motil..

[46] Faure C., Wieckowska A. (2007). Somatic referral of visceral sensations and rectal sensory threshold for pain in children with functional gastrointestinal disorders. J. Pediatr..

[47] DiLorenzo C., Youssef N.N., Sigurdsson L., Scharff L., Griffiths J., Wald A. (2001). Visceral hyperalgesia in children with functional abdominal pain. J. Pediatr..

[48] Collins S.M., Piche T., Rampal P. (2001). The putative role of inflammation in the irritable bowel syndrome. Gut.

[49] Cremon C., Gargano L., Morselli-Labate A.M., Santini D., Cogliandro R.F., De Giorgio R., Stanghellini V., Corinaldesi R., Barbara G. (2009). Mucosal immune activation in irritable bowel syndrome: Gender-dependence and association with digestive symptoms. Am. J. Gastroenterol..

[50] Anhe F.F., Roy D., Pilon G., Dudonne S., Matamoros S., Varin T.V., Garofalo C., Moine Q., Desjardins Y., Levy E. (2015). A polyphenol-rich cranberry extract protects from diet-induced obesity, insulin resistance and intestinal inflammation in association with increased Akkermansia spp. population in the gut microbiota of mice. Gut.

[51] Sundin J., Rangel I., Repsilber D., Brummer R.J. (2015). Cytokine Response after Stimulation with Key Commensal Bacteria Differ in Post-Infectious Irritable Bowel Syndrome (PI-IBS) Patients Compared to Healthy Controls. PLoS ONE.

[52] Hippe B., Remely M., Bartosiewicz N., Riedel M., Nichterl C., Schatz L., Pummer S., Haslberger A. (2014). Abundance and diversity of GI microbiota rather than IgG4 levels correlate with abdominal inconvenience and gut permeability in consumers claiming food intolerances. Endocrine Metab. Immune Disord. Drug Targets.

[53] Kamp K.J., Plantinga A.M., Cain K.C., Burr R.L., Barney P., Jarrett M., Luna R.A., Savidge T., Shulman R., Heitkemper M.M. (2021). A Comprehensive Self-Management Program With Diet Education Does Not Alter Microbiome Characteristics in Women with Irritable Bowel Syndrome. Biol. Res. Nurs..

[54] Jacobs J.P., Gupta A., Bhatt R.R., Brawer J., Gao K., Tillisch K., Lagishetty V., Firth R., Gudleski G.D., Ellingson B.M. (2021). Cognitive behavioral therapy for irritable bowel syndrome induces bidirectional alterations in the brain-gut-microbiome axis associated with gastrointestinal symptom improvement. Microbiome.

[55] Chumpitazi B.P., Hoffman K.L., Smith D.P., McMeans A.R., Musaad S., Versalovic J., Petrosino J., Shulman R.J. (2021). Fructan-sensitive children with irritable bowel syndrome have distinct gut microbiome signatures. Aliment. Pharmacol. Ther..

[56] Hollister E.B., Oezguen N., Chumpitazi B.P., Luna R.A., Weidler E.M., Rubio-Gonzales M., Dahdouli M., Cope J.L., Mistretta T.A., Raza S. (2019). Leveraging Human Microbiome Features to Diagnose and Stratify Children with Irritable Bowel Syndrome. J. Mol. Diagn..

[57] Bonilla S., Saps M. (2013). Early life events predispose the onset of childhood functional gastrointestinal disorders. Rev. Gastroenterol. Mexico.

[58] Chiou E., Nurko S. (2011). Functional abdominal pain and irritable bowel syndrome in children and adolescents. Therapy.

[59] Levy R.L., van Tilburg M.A.L. (2012). Functional abdominal pain in childhood: Background studies and recent research trends. Pain Res. Manag..

[60] Langer S.L., Romano J.M., Levy R.L., Walker L.S., Whitehead W.E. (2009). Catastrophizing and parental response to child symptom complaints. Child Health Care.

[61] Eccleston C., Palermo T.M., de C Williams A.C., Lewandowski A., Morley S., Fisher E., Law E. (2012). Psychological therapies for the management of chronic and recurrent pain in children and adolescents. Cochrane Database Syst. Rev..

[62] Sprenger L., Gerhards F., Goldbeck L. (2011). Effects of psychological treatment on recurrent abdominal pain in children—A meta-analysis. Clin. Psychol. Rev..

[63] Palermo T.M., Eccleston C., Lewandowski A., de C Williams A.C., Morley S. (2010). Randomized controlled trials of psychological therapies for management of chronic pain in children and adolescents: An updated meta-analytic review. Pain.

[64] Ford A.C., Lacy B.E., Harris L.A., Quigley E.M.M., Moayyedi P. (2019). Effect of Antidepressants and Psychological Therapies in Irritable Bowel Syndrome: An Updated Systematic Review and Meta-Analysis. Am. J. Gastroenterol..

[65] Lackner J.M., Jaccard J., Krasner S.S., Katz L.A., Gudleski G.D., Blanchard E.B. (2007). How does cognitive behavior therapy for irritable bowel syndrome work? A mediational analysis of a randomized clinical trial. Gastroenterology.

[66] Ford A.C., Quigley E.M., Lacy B.E., Lembo A.J., Saito Y.A., Schiller L.R., Soffer E.E., Spiegel B.M., Moayyedi P. (2014). Effect of antidepressants and psychological therapies, including hypnotherapy, in irritable bowel syndrome: Systematic review and meta-analysis. Am. J. Gastroenterol..

[67] Abbott R.A., Martin A.E., Newlove-Delgado T.V., Bethel A., Thompson-Coon J., Whear R., Logan S. (2017). Psychosocial interventions for recurrent abdominal pain in childhood. Cochrane Database Syst. Rev..

[68] Fisher E., Law E., Palermo T.M., Eccleston C. (2015). Psychological therapies (remotely delivered) for the management of chronic and recurrent pain in children and adolescents. Cochrane Database Syst. Rev..

[69] Levy R.L., Langer S.L., Walker L.S., Romano J.M., Christie D.L., Youssef N., DuPen M.M., Feld A.D., Ballard S.A., Welsh E.M. (2010). Cognitive-behavioral therapy for children with functional abdominal pain and their parents decreases pain and other symptoms. Am. J. Gastroenterol..

[70] Levy R.L., Langer S.L., van Tilburg M.A., Romano J.M., Murphy T.B., Walker L.S., Mancl L.A., Claar R.L., DuPen M.M., Whitehead W.E. (2017). Brief telephone-delivered cognitive behavioral therapy targeted to parents of children with functional abdominal pain: A randomized controlled trial. Pain.

[71] van Tilburg M.A., Claar R.L., Romano J.M., Langer S.L., Walker L.S., Whitehead W.E., Abdullah B., Christie D.L., Levy R.L. (2015). Role of coping with symptoms in depression and disability: Comparison between inflammatory bowel disease and abdominal pain. J. Pediatr. Gastroenterol. Nutr..

[72] Levy R.L., Whitehead W.E., Walker L.S., Von K.M., Feld A.D., Garner M., Christie D. (2004). Increased somatic complaints and health-care utilization in children: Effects of parent IBS status and parent response to gastrointestinal symptoms. Am. J. Gastroenterol..

[73] Cunningham N.R., Lynch-Jordan A., Barnett K., Peugh J., Sil S., Goldschneider K., Kashikar-Zuck S. (2014). Child pain catastrophizing mediates the relation between parent responses to pain and disability in youth with functional abdominal pain. J. Pediatr. Gastroenterol. Nutr..

[74] Levy R.L., Langer S., Walker L., Romano J., Christie D., Youssef N., DuPen M., Ballard S., Labus J., Welsh E. (2013). Twelve month follow-up of cognitive behavioral therapy for children with functional abdominal pain. JAMA Pediatr..

[75] Laird K.T., Tanner-Smith E.E., Russell A.C., Hollon S.D., Walker L.S. (2016). Short-term and Long-term Efficacy of Psychological Therapies for Irritable Bowel Syndrome: A Systematic Review and Meta-analysis. Clin. Gastroenterol. Hepatol..

[76] Ljotsson B., Andersson E., Lindfors P., Lackner J.M., Gronberg K., Molin K., Noren J., Romberg K., Andersson E., Hursti T. (2013). Prediction of symptomatic improvement after exposure-based treatment for irritable bowel syndrome. BMC Gastroenterol..

[77] Martin A.E., Newlove-Delgado T.V., Abbott R.A., Bethel A., Thompson-Coon J., Whear R., Logan S. (2017). Pharmacological interventions for recurrent abdominal pain in childhood. Cochrane Database Syst. Rev..

[78] Rodriguez-Fandino O., Hernandez-Ruiz J., Schmulson M. (2010). From cytokines to toll-like receptors and beyond—Current knowledge and future research needs in irritable bowel syndrome. J. Neurogastroenterol. Motil..

[79] Mayer E.A., Savidge T., Shulman R.J. (2014). Brain-gut microbiome interactions and functional bowel disorders. Gastroenterology.

[80] Labus J.S. (2007). In search of mechanisms of change in treatment outcome research: Mediators and moderators of psychological and pharmacological treatments for irritable bowel syndrome. Gastroenterology.

[81] Jarrett M.E., Cain K.C., Barney P.G., Burr R.L., Naliboff B.D., Shulman R., Zia J., Heitkemper M.M. (2016). Balance of Autonomic Nervous System Predicts Who Benefits from a Self-management Intervention Program for Irritable Bowel Syndrome. J. Neurogastroenterol. Motil..

[82] Levy R.L., van Tilburg M., Langer S.L., Romano J., Mancl L., Whitehead W.E., Feld S., Walker L.S. (2015). Parent-only intervention reduces symptoms and disability in abdominal pain patients. J. Pediatr. Gastroenterol. Nutr..

[83] Levy R.L., van Tilburg M.A., Langer S.L., Romano J.M., Walker L.S., Mancl L.A., Murphy T.B., Claar R.L., Feld S.I., Christie D.L. (2016). Effects of a cognitive behavioral therapy intervention trial to improve disease outcomes in children with Inflammatory Bowel Disease. Inflamm. Bowel Dis..

[84] Walker L.S., Garber J., Van Slyke D.A. (1995). Do parents excuse the misbehavior of children with physical or emotional symptoms? An investigation of the pediatric sick role. J. Pediatr. Psychol..

[85] van Tilburg M.A.L., Venepalli N., Ulshen M., Freeman K.L., Levy R., Whitehead W.E. (2006). Parents’ worries about recurrent abdominal pain in children. Gastroenterol. Nurs..

[86] van Tilburg M.A.L., Chitkara D.K., Palsson O.S., Turner M., Blois-Martin N., Ulshen M., Whitehead W.E. (2009). Audio-recorded guided imagery treatment reduces functional abdominal pain in children: A pilot study. Pediatrics.

[87] Levy R.L. (1983). Social support and compliance: A selective review and critique of treatment integrity and outcome measurement. Soc. Sci. Med..

[88] Barrett J.S. (2013). Extending our knowledge of fermentable, short-chain carbohydrates for managing gastrointestinal symptoms. Nutr. Clin. Pract..

[89] O’Brien L., Kasti A., Halmos E.P., Tuck C.J., Varney J. (2024). Evolution, adaptation, and new applications of the FODMAP diet. JGH Open.

[90] Sultan N., Varney J.E., Halmos E.P., Biesiekierski J.R., Yao C.K., Muir J.G., Gibson P.R., Tuck C.J. (2022). How to implement the 3-phase FODMAP diet into gastroenterological practice. J. Neurogastroenterol. Motil..

[91] Liu J., Chey W.D., Haller E., Eswaran S. (2020). Low-FODMAP Diet for Irritable Bowel Syndrome: What We Know and What We Have Yet to Learn. Annu. Rev. Med..

[92] Chumpitazi B.P., Weidler E.M., Shulman R.J. (2011). A Multi-Substrate Carbohydrate Elimination Diet (MCED) Decreases Gastrointestinal (GI) Symptoms in a Subpopulation of Children With Irritable Bowel Syndrome (IBS). Gastroenterology.

[93] Harris P.A., Taylor R., Thielke R., Payne J., Gonzalez N., Conde J.G. (2009). Research electronic data capture (REDCap)—A metadata-driven methodology and workflow process for providing translational research informatics support. J. Biomed. Inform..

[94] Harris P.A., Taylor R., Minor B.L., Elliott V., Fernandez M., O’Neal L., McLeod L., Delacqua G., Delacqua F., Kirby J. (2019). The REDCap consortium: Building an international community of software platform partners. J. Biomed. Inform..

[95] Rockett H.R., Breitenbach M., Frazier A.L., Witschi J., Wolf A.M., Field A.E., Colditz G.A. (1997). Validation of a youth/adolescent food frequency questionnaire. Prev. Med..

[96] Von Baeyer C.L., Spagrud L.J., McCormick J.C., Choo E., Neville K., Connelly M.A. (2009). Three new datasets supporting use of the Numerical Rating Scale (NRS-11) for children’s self-reports of pain intensity. Pain.

[97] Wong D.I., Baker C.M. (1988). Pain in children: Comparison of assessment scales. Pediatr. Nurs..

[98] Varni J.W., Burwinkle T.M., Seid M., Skarr D. (2003). The PedsQL 4.0 as a pediatric population health measure: Feasibility, reliability, and validity. Ambul. Pediatr..

[99] Varni J.W., Seid M., Kurtin P.S. (2001). PedsQL 4.0: Reliability and validity of the Pediatric Quality of Life Inventory Version 4.0 Generic Core Scales in healthy and patient populations. Med. Care.

[100] Varni J.W., Shulman R.J., Self M.M., Nurko S., Saps M., Saeed S.A., Bendo C.B., Patel A.S., Dark C.V., Zacur G.M. (2015). Symptom Profiles in Patients With Irritable Bowel Syndrome or Functional Abdominal Pain Compared With Healthy Controls. J. Pediatr. Gastroenterol. Nutr..

[101] Owens J.A., Spirito A., McGuinn M. (2000). The Children’s Sleep Habits Questionnaire (CSHQ): Psychometric properties of a survey instrument for school-aged children. Sleep.

[102] Schurman J.V., Friesen C.A., Dai H., Danda C.E., Hyman P.E., Cocjin J.T. (2012). Sleep problems and functional disability in children with functional gastrointestinal disorders: An examination of the potential mediating effects of physical and emotional symptoms. BMC Gastroenterol..

[103] Sandercock G.R., Bromley P.D., Brodie D.A. (2005). The reliability of short-term measurements of heart rate variability. Int. J. Cardiol..

[104] Jarrett M.E., Burr R.L., Cain K.C., Hertig V., Weisman P., Heitkemper M.M. (2003). Anxiety and depression are related to autonomic nervous system function in women with irritable bowel syndrome. Dig. Dis. Sci..

[105] Jarrett M.E., Burr R.L., Cain K.C., Rothermel J.D., Landis C.A., Heitkemper M.M. (2008). Autonomic nervous system function during sleep among women with irritable bowel syndrome. Dig. Dis. Sci..

[106] McOmber M.E., Ou C.N., Shulman R.J. (2010). Effects of timing, sex, and age on site-specific gastrointestinal permeability testing in children and adults. J. Pediatr. Gastroenterol. Nutr..

[107] Meddings J.B., Gibbons I. (1998). Descrimination of site-specific alterations in gastrointestinal permeability in the rat. Gastroenterology.

[108] Bjarnason I., MacPherson A., Hollander D. (1995). Intestinal permeability: An overview. Gastroenterology.

[109] Hollander D., Vadheim C.M., Brettholz E., Petersen G.M., Delahunty T., Rotter J.I. (1986). Increased intestinal permeability in patients with Crohn’s disease and their relatives. A possible etiologic factor. Ann. Intern. Med..

[110] Saulnier D.M., Ringel Y., Heyman M.B., Foster J.A., Bercik P., Shulman R.J., Versalovic J., Verdu E.F., Dinan T.G., Hecht G. (2013). The intestinal microbiome, probiotics and prebiotics in neurogastroenterology. Gut Microbes.

[111] Aagaard K., Petrosino J., Keitel W., Watson M., Katancik J., Garcia N., Patel S., Cutting M., Madden T., Hamilton H. (2013). The Human Microbiome Project strategy for comprehensive sampling of the human microbiome and why it matters. FASEB J.

[112] Ohman L., Stridsberg M., Isaksson S., Jerlstad P., Simren M. (2012). Altered levels of fecal chromogranins and secretogranins in IBS: Relevance for pathophysiology and symptoms?. Am. J. Gastroenterol..

[113] Shulman R.J., Ohman L., Stridsberg M., Cain K., Simren M., Heitkemper M. (2019). Evidence of increased fecal granins in children with irritable bowel syndrome and correlates with symptoms. Neurogastroenterol. Motil..

[114] Walker L.S., Smith C.A., Garber J., Van Slyke D.A. (1997). Development and validation of the pain response inventory for children. Psych. Assess..

[115] Derogatis L.R. (1994). Symptom Checklist-90-R: Administrative Scoring and Procedures Manual.

[116] Derogatis L.R., Savitz K.L., Maruish M.E. (2000). The SCL-90-R, Brief Symptom Inventory, and Matching Clinical Rating Scales. The Use of Psychological Testing for Treatment Planning and Outcomes Assessment.

[117] Crombez G., Bijttebier P., Eccleston C., Mascagni T., Mertens G., Goubert L., Verstraeten K. (2003). The child version of the pain catastrophizing scale (PCS-C): A preliminary validation. Pain.

[118] Sullivan M.J.L., Bishop S.R., Pivik J. (1995). The pain catastrophizing scale: Development and validation. Psychol. Assess..

[119] Pielech M., Ryan M., Logan D.E., Kaczynski K., White M.T., Simons L.E. (2014). Pain catastrophizing in children with chronic pain and their parents: Proposed clinical reference points and re-examination of the Pain Catastrophizing Scale measure. Pain.

[120] Parkerson H.A., Noel M., Page M.G., Fuss S., Katz J., Asmundson G.J. (2013). Factorial validity of the English-language version of the Pain Catastrophizing Scale—Child version. J. Pain.

[121] Kerns R.D., Haythornthwaite J., Rosenberg R., Southwick S., Giller E.L., Jacob M.C. (1991). The Pain Behavior Check List (PBCL): Factor structure and psychometric properties. J. Behav. Med..

[122] Marshall W.A., Tanner J.M. (1970). Variations in the pattern of pubertal changes in boys. Arch. Dis. Child.

[123] Marshall W.A., Tanner J.M. (1969). Variations in the pattern of pubertal changes in girls. Arch. Dis. Child.

[124] Doyle A., Ostrander R., Skare S., Crosby R.D., August G.J. (1997). Convergent and criterion-related validity of the Behavior Assesment System for Children-Parent Rating Scale. J. Clin. Child Psychol..

[125] Deighton J., Croudace T., Fonagy P., Brown J., Patalay P., Wolpert M. (2014). Measuring mental health and wellbeing outcomes for children and adolescents to inform practice nad policy: A review of child self-report measures. Child Adolesc. Psychiatry Ment. Health.

[126] Owens J.A., Spirito A., McGuinn M., Nobile C. (2000). Sleep habits and sleep disturbance in elementary school-aged children. J. Dev. Behav. Pediatr..

[127] Palsson O.S., Whitehead W.E., van Tilburg M.A., Chang L., Chey W., Crowell M.D., Keefer L., Lembo A.J., Parkman H.P., Rao S.S. (2016). Rome IV Diagnostic Questionnaires and Tables for Investigators and Clinicians. Gastroenterology.

[128] Walker L.S., Levy R.L., Whitehead W.E. (2006). Validation of a measure of protective parent responses to children’s pain. Clin. J. Pain.

[129] Walker L.S., Baber K.F., Garber J., Smith C.A. (2008). A typology of pain coping strategies in pediatric patients with chronic abdominal pain. Pain.

[130] Walker L.S., Smith C.A., Garber J., Claar R.L. (2005). Testing a model of pain appraisal and coping in children with chronic abdominal pain. Health Psychol..

[131] Walker L.S., Greene J.W. (1991). The functional disability inventory: Measuring a neglected dimension of child health status. J. Pediatr. Psychol..

[132] Sole E., Galan S., de la Vega R., Castarlenas E., Sanchez-Rodriguez E., Jensen M.P., Miro J. (2019). Psychometric properties of the Functional Disability Inventory for assessing pain-related disability in children from the community. Disabil. Rehabil..

[133] Stahlschmidt L., Friedrich Y., Zernikow B., Wager J. (2018). Assessment of pain-related disability in pediatric chronic pain: A comparison of the Functional Disability Inventory and the Pediatric Pain Disability Index. Clin. J. Pain.

[134] Kashikar-Zuck S., Flowers S.R., Claar R.L., Guite J.W., Logan D.E., Lynch-Jordan A.M., Palermo T.M., Wilson A.C. (2011). Clinical utility and validity of the Functional Disability Inventory among a multicenter sample of youth with chronic pain. Pain.

