# Supplementary figures and images for: Protocol for a Randomized Controlled Trial to Determine if Biomarkers Predict Response to a Pediatric Chronic Pain Symptom Management Program
Source: J Clin Med. 2025 May 5;14(9):3185. doi: 10.3390/jcm14093185 (PMC12072368; doi:10.3390/jcm14093185)

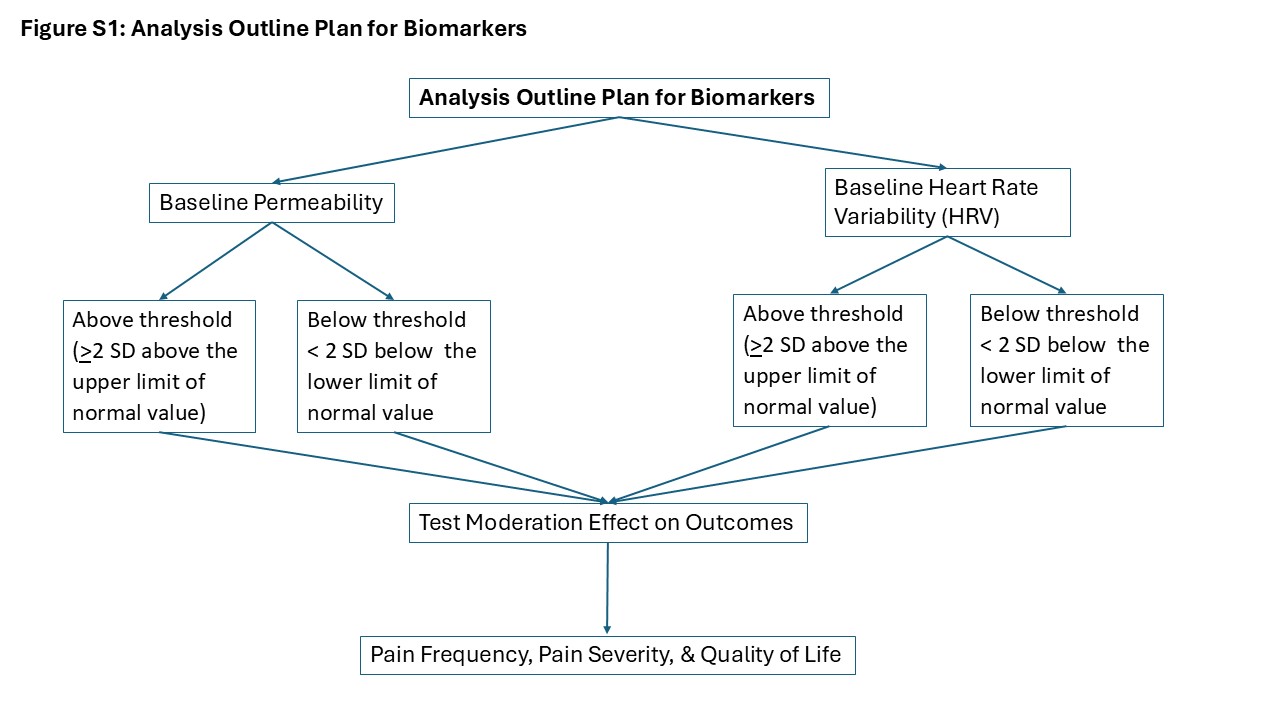

Supplement: Supplementary file 1 [file jcm-14-03185-s001.zip › jcm-3505295-supplementary.jpg]
